# Mixed neuroendocrine and squamous cell carcinoma of the colon: A case report and literature review

**DOI:** 10.1016/j.ijscr.2019.06.060

**Published:** 2019-06-28

**Authors:** Adel Elkbuli, Brianna Dowd, Mark McKenney, Dessy Boneva

**Affiliations:** a Department of Surgery, Kendall Regional Medical Center, Miami, FL, United States; b Department of Surgery, University of South Florida, Tampa, FL, United States

**Keywords:** Neuroendocrine carcinoma, Squamous cell carcinoma, Mixed neuroendocrine and squamous cell carcinoma, Primary colon cancer, Case report

## Abstract

**Introduction:**

The occurrence of Neuroendocrine Carcinoma (NEC) of the colon is <2% of all colon cancers. Squamous Cell Carcinoma (SCC) is even more unusual and has an occurrence in the colon as low as 0.1% of colon cancers. Coupled together mixed NEC-SCC, is nearly unheard of. SCC of the gastrointestinal tract is usually found in the esophagus and rectum, rarely are there squamous cells even present in the colon.

**Presentation of case:**

A 62-year-old female presented with abdominal pain and a palpable mass in the left lower quadrant. CT scan revealed a poly-lobulated mass in the left abdomen attached to the left colon. A left hemicolectomy was performed. There were no distant metastases at the time of diagnosis. Microscopic examination revealed NEC mixed with SCC demonstrated by keratin pearl formation.

**Discussion:**

NEC and SCC individually are unusual in the colon but together are extraordinarily rare. Histologically, the NEC in this case demonstrated squamous cell differentiation with keratin pearl formation. The median survival in studies of patients with colonic NEC is 5–10 months due to the aggressive behavior and lymph node metastases, which were present in this case. There is insufficient literature addressing ideal adjuvant therapy after resection of mixed NEC and SCC of the colon.

**Conclusion:**

We present the first case of primary colon mixed NEC and SCC which presented as abdominal pain. There is a current absence of ideal therapy recommendations in the medical literature following resection of mixed NEC and SCC of the colon.

## Highlights

• We present the first case of primary colon mixed NEC and SCC which presented with abdominal pain.
• The tumor was resected, and patient was set to start chemotherapy treatment.
• There is not sufficient literature addressing ideal adjuvant therapy after resection of mixed NEC and SCC.

## 1 Introduction

Colorectal cancer is the third most common cancer, excluding skin cancers, and will have an estimated incidence of over 100,000 new cases of in 2019 [1]. The most commonly found carcinomas of the colon are adenocarcinomas [2]. Alternatively, Neuroendocrine Carcinoma (NEC) of the colon is an unusual type of cancer that occurs in the endocrine epithelial cells. The occurrence of NEC of the colon is <2% of all colorectal malignancies [3]. NEC is an aggressive carcinoma due to lymph node metastases. There are endocrine markers expressed on the tumor cells which make up NEC, allowing for identification and diagnosis. Squamous cell carcinoma (SCC) typically involves the esophagus or anus, rarely the colon. SCC has an occurrence of 0.1-0.25% of cases of colorectal carcinoma [4]. SCC of the colon has less than 100 cases reported in the literature [5]. The pathogenesis of SCC is unclear, and a commonly reported theory suggests that multipotent cells differentiate into squamous cells from mucosal injury [2,5]. SCC has been seen more commonly in the colon as “pure” squamous cells [6], but rarely is SCC seen as mixed with another epithelium carcinoma.

Herein, we present the first case of primary colon mixed NEC and SCC to be reported in the literature. The tumor was resected, and the patient was set to start chemotherapy treatment; however, there is not sufficient literature addressing ideal adjuvant therapy after resection of mixed NEC and SCC of the colon. This work has been reported in line with the SCARE criteria [7].

## 2 Presentation of case

A 62-year-old female was admitted to our Emergency Department with a chief complaint of left lower quadrant abdominal pain. The patient also complained of not having had bowel movements for several days before coming to the hospital. She had a history of hypertension controlled with medication she did not smoke and had no previous surgical or hospitalization history.

**Fig. 1:**
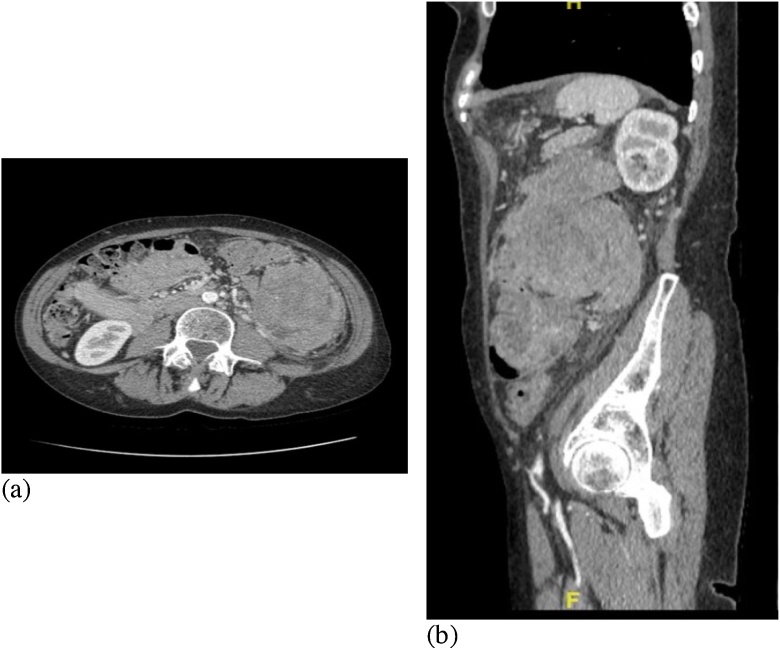
CT Scans with IV contrast; (a) shows the axial view revealing a large polylobulated mass in the left abdomen, and (b) shows the sagittal view revealing a large mass occupying most of the left abdomen.

A mass was palpable in the lower left quadrant. Computed tomography (CT) of the abdomen and pelvis revealed a poly-lobulated mass in the left abdomen emanating from the left colon (Fig. 1). Small to moderate pockets of free fluid in the abdomen and pelvis were observed. Moderate-to-large amount of stool in the proximal colon suggested constipation and partial obstruction (Fig. 1). However, there was no significant bowel dilatation.

**Fig. 2:**
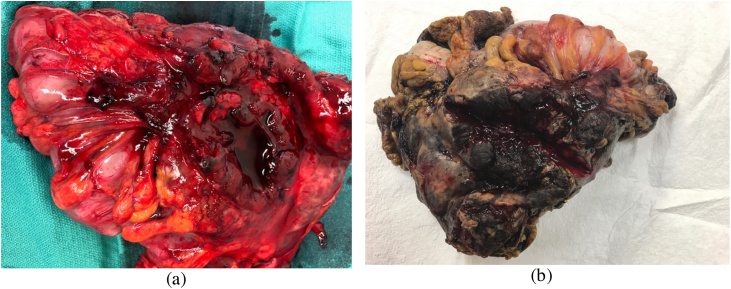
Resected Specimen shows (a) the left colon mass immediately upon resection measured at 20 x 23 cm, and (b) the resected tumor during analysis.

**Fig. 3:**
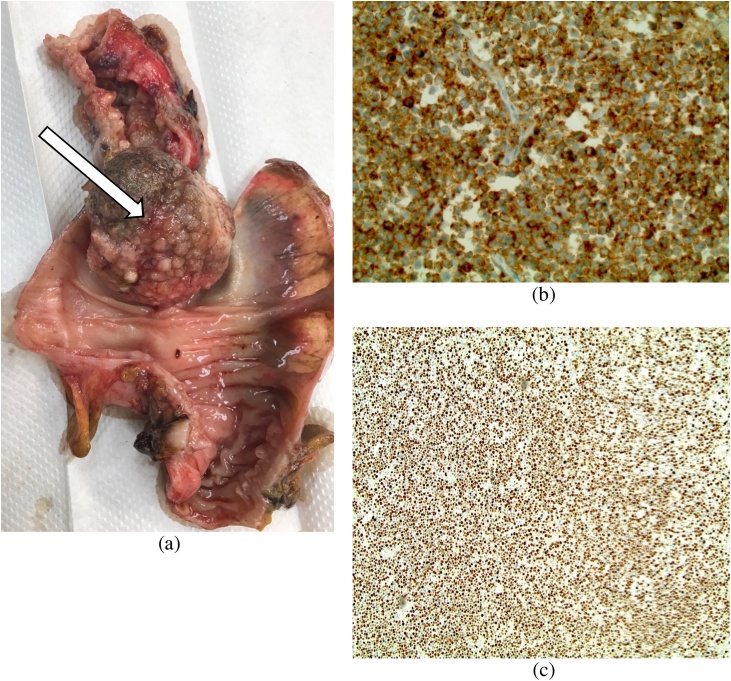
Neuroendocrine Carcinoma shows (a) the isolated spherical tumor obstructing the colon lumen, which is characteristic of neuroendocrine tumors, (b) the cells labeled with the synaptophysin stain at 100X, which strongly demonstrates neuroendocrine carcinoma, and (c) the proliferation marker Ki-67 labelling approaching 100%, which suggests malignant tissue with poorly differentiated tumor cells.

Colonoscopy revealed an obstructing tumor in the descending colon. During colonoscopy, superficial biopsies were taken which showed villous adenoma on top of the tumor. The patient was taken to the operating theater and underwent a left hemicolectomy with primary anastomosis of the colon without diverting ostomy. Upon surgical intervention, the tumor extended through the colon wall with pericolonic hemorrhage (Fig. 2) arising from the mesocolon. The pathologic evaluation revealed extension of the tumor with metastasis to local lymph nodes and with hemorrhage. The mass measured 7 cm in greatest dimension which on section was nodular and brown (Fig. 2, Fig. 3). Tumor markers were elevated, including: cancer antigen 125 (CA125) at 165 (0–32 mg/ml). Cancer Antigen 19-9 (CA19-9) was also elevated at 48 (0–31 U/ml). Postoperatively the patient did well, diet was advanced and she was discharged five days later. Two weeks later in the office visit, she was doing well.

**Fig. 4:**
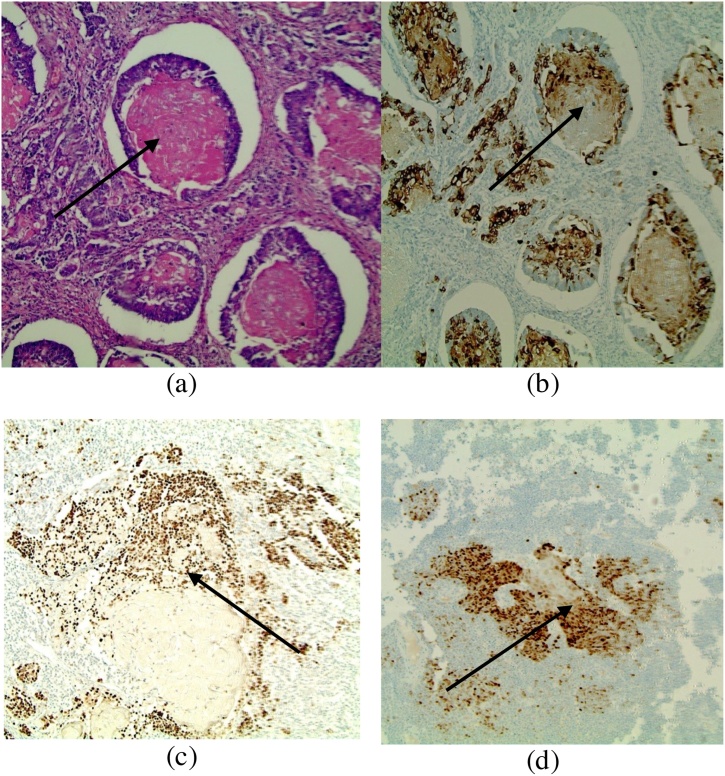
Squamous Cell Carcinoma shows (a) the characteristic keratin pearls of squamous cell carcinoma in the tumor, (b) the squamous cells labeled with the cytokeratin marker 5/6 (CK 5/6) at 100X, (c) the squamous cells labeled with the P63 antibody marker at 40X, and (d) the squamous cells labeled with the CDX2 marker at 40X, which show intense positivity.

Microscopically, the tumor was composed of cells with small nuclei and scant cytoplasm, having the appearance of lymphocytes. They were arranged in large packets – separated by fine fibrovascular septa, so there is an organoid architecture, but it is very subtle. Nuclear molding was not observed. A CD45 (lymphocyte common antigen) IHC stain was negative. There were also nests of cells with a squamous appearance with keratin pearl formation. The proliferation marker Ki-67 was stained and was almost 100% (Fig. 3). The squamous cells were positive for cytokeratin marker 5/6 (CK5/6), P40 marker and P63 marker (Fig. 4). These markers confirm squamous cell differentiation. Interestingly they were also positive for CDX2 staining (Fig. 4), which indicates these cells have intestinal differentiation. The lymph node metastases showed similar features to that in the colonic mass with both neuroendocrine and squamous cell features.

The final diagnosis was infiltrating large-cell neuroendocrine carcinoma with squamous cell carcinoma of the sigmoid colon. The histologic grade was G3, poorly differentiated.

## 3 Discussion

The rare incidence of mixed NEC and SCC of the colon yields it a largely unknown cancer. Nevertheless, the World Health Organization has classified neoplasms of the digestive system in 2010 with neuroendocrine differentiation into neuroendocrine tumor (NET), neuroendocrine carcinoma (NEC), and mixed adeno-neuroendocrine carcinoma (MANEC) [8]. Though a diagnosis of MANEC can only be done when there are both neuroendocrine and non-neuroendocrine epithelial malignant components and each component is at least 30% of the tumor [8]. SCC is classified as either in situ or invasive, meaning the carcinoma is locally confined or has invaded adjacent structures respectively. There is limited information on the classification of mixed NEC and SCC, with no cases reported in the literature on specifically colon primary mixed NEC and SCC – like our case. We describe the first case of primary colon mixed NEC and SCC.

As was seen in this case, the majority of patients with NET present with abdominal pain. Tumors are often identified by colonoscopy. Similarly, symptoms of SCC of the colon have been clinically compared to that of adenocarcinoma [9], which includes constipation, rectal bleeding, and abdominal pain. Our patient showed symptoms of abdominal pain and constipation. Abdominal imaging (CT or MRI) combined with colonoscopy can provide sufficient information to diagnose NET, which can be confirmed with immunohistochemical staining. These carcinomas have a poor prognosis due to their aggressive behavior. The median survival in studies of patients with colonic neuroendocrine carcinoma is 5–10 months [3,10]. This is due to the high rate of lymph node metastases as was seen in our case. Patients have been shown to have a better prognosis if they present without distant metastatic disease [11]. Distant metastases in our case were not found. The Ki-67 (Fig. 3) being almost 100% accounts for the tumor’s aggressive behavior.

Munakata et al. recently published a case report of mixed neuroendocrine carcinoma and squamous cell carcinoma of the lung that metastasized to the colon and liver, which was treated with six cycles of chemotherapy, but the patient was not eligible for additional chemotherapy after 2 months [12]. The authors suggest a possible common link between the pathogenesis and conclude future research is required to determine the pathogenesis of these synchronous tumors [12]. Our case alternatively reports primary colon cancer with no distant metastases at the time of diagnosis, but rather local lymph and tissue metastases which were excised.

Further, Vardas et al. published a case report of mixed large cell neuroendocrine carcinoma with squamous cell carcinoma of the rectum which, after refusal of chemotherapy, resulted in the patient’s death by liver failure as a result of multiple hepatic metastases [13]. The authors conclude that low anterior resection and total mesorectal excision with free surgical margins in the presence of lymph nodes metastasis is not a sufficient treatment for rectal neuroendocrine carcinoma [13]. Unlike our case in which the mixed NEC and SCC are located in the colon, this case presents such cancer located in the rectum. This case is more explainable than ours because SCC of the gastrointestinal tract is usually found in the esophagus and rectum. This raises the question as to how NEC became mixed with SCC in our case.

It is thought that the bidirectional differentiation is due to the neuroendocrine cells having acquired an APUD phenotype and arising from endoderm stem cells. It has been proposed that colonic stem cells are central to the initiation of cancer. The expression pattern of colorectal stem cells in colon adenocarcinoma reveals a heterogeneous population of cells ranging from pluripotent to differentiated cells with overlapping and sometimes unique combinations. These observations might explain the differing morphologies and staining found in our case. Other studies have suggested that inflammatory diseases may cause squamous metaplasia, from which carcinoma then develops [14].

There is not sufficient literature addressing ideal adjuvant therapy after resection of mixed NEC and SCC of the colon. The preferred approach in patients with a localized tumor or a tumor spread to regional lymph nodes is surgical resection [14]. It is suggested that advanced GI-NEC patients be considered for chemotherapy treatment without delay [15]. The role and benefits of systemic adjuvant therapy or of neoadjuvant or adjuvant chemoradiotherapy for patients with mixed NEC and SCC remain unknown [15]. Additionally, the literature suggests no optimal follow-up for these patients.

## 4 Conclusion

We present the first case of primary colon mixed NEC and SCC which presented as abdominal pain. There is a current absence of ideal therapy recommendations in the medical literature following resection of mixed NEC and SCC of the colon.

## Conflicts of interest

None.

## Funding

None.

## Ethical approval

This is a case report study. Informed written consent has been obtained and all identifying information is omitted. This work has been conducted in compliance with institutional ethical standards.

## Consent

Informed written consent has been obtained and all identifying information is omitted.

## Author contribution

Adel Elkbuli, Brianna Dowd, Dessy Boneva, Mark McKenney– Conception of study, acquisition of data, analysis and interpretation of data.

Adel Elkbuli, Dessy Boneva, Brianna Dowd - Drafting the article.

Dessy Boneva, Mark McKenney – Management of case.

Adel Elkbuli, Brianna Dowd, Dessy Boneva, Mark McKenney – Critical revision of article and final approval of the version to be submitted.

## Registration of research studies

This is a case report study.

## Guarantor

Dessy Boneva.

Mark McKenney.

## Provenance and peer review

Not commissioned, externally peer-reviewed.
